# Unmet need for contraception and associated factors among women with cardiovascular disease having follow-up at Saint Paul’s Hospital Millennium Medical College, Addis Ababa, Ethiopia: a cross-sectional study

**DOI:** 10.1186/s40834-022-00173-0

**Published:** 2022-05-11

**Authors:** Negalign Mechal, Mustefa Negash, Hailemichael Bizuneh, Ferid A. Abubeker

**Affiliations:** 1 Department of Obstetrics and Gynecology, Saint Paul’s Hospital Millennium Medical College, Addis Ababa, Ethiopia; 2 Epidemiology Unit, Department of Public Health, Saint Paul’s Hospital Millennium Medical College, Addis Ababa, Ethiopia

**Keywords:** Unmet need, Contraception, Cardiovascular disease, Chronic medical disease, Ethiopia

## Abstract

**Background:**

Unmet need for contraception refers to the proportion of women who want to postpone or stop childbearing but are not using a contraceptive method. Addressing unmet need is especially important for women with medical conditions such as cardiovascular disease (CVD). Preventing unintended pregnancy is crucial to improve pregnancy outcomes and minimize complications of CVD during pregnancy. However, unmet need for contraceptives continues to undermine the potential benefits of contraceptive use. This research aimed to determine the rate of unmet need for contraceptives and associated factors among women with cardiovascular disease having follow-up at Saint Paul’s Hospital Millennium Medical College (SPHMMC), Addis Ababa, Ethiopia.

**Methods:**

A facility-based cross-sectional study was conducted from February 1 to May 31/2020. A total of 284 reproductive age women with cardiovascular disease having follow-up at the cardiac clinic of SPHMMC were enrolled consecutively until the desired sample size was reached. Data was collected through an exit interview using a structured and pretested questionnaire. Descriptive, bivariate, and multivariable methods were used to analyze the level of unmet need and its associated factors.

**Results:**

The overall unmet need for contraception was 36.0% (95% CI: 30.4–41.5). The majority of the respondents lack counseling on contraception use. The most common reasons for non-use of a contraceptive method were fear of drug side effects and drug interaction. Unmet need for contraception was found to be more likely among those who have not been counseled on contraceptive utilization (AOR 6.7, CI 1.8–24.7) and those who lack partner support on contraception use (AOR = 6.2, CI: 1.91–19.8). Unmet need was also found to be more likely among women who have never used contraception before (AOR = 3.2, CI 1.12–8.92).

**Conclusion:**

Unmet need for contraception was high in this high-risk population group. The cardiac follow-up clinic should implement client-centered counseling by a multidisciplinary team to address the needs of women and prevent consequences of unintended pregnancy. Furthermore, there is a need to initiate interventions that encourage communication between couples and increase male partner involvement through a renewed focus on couples counseling.

**Supplementary Information:**

The online version contains supplementary material available at 10.1186/s40834-022-00173-0.

## Background

Unmet need for contraception refers to the proportion of women who want to either postpone the next birth for at least 2 years (spacers) or stop childbearing (limiters) but are not using a contraceptive method. Estimates of unmet need along with the proportion of women currently using contraception are used to determine the total demand for contraception and the demand satisfied [1, 2]. These indicators are widely used in policy development, implementation, and monitoring of family planning programs and provide a means to compare trends across settings and over time and evaluate the impact of interventions [3, 4].

Several global initiatives attempted to address unmet need and substantial progress has been made in the last 20 years. Between 2000 and 2020, the number of women using contraceptive methods increased by 188 million worldwide and the demand satisfied for contraceptives has increased from 73.6% to 76.8%. Since 2000 the level of unmet need has been declining progressively in all regions. In 2020, 10% of women worldwide had unmet need. However, great disparities remain across regions. Unmet need is highest in sub-Saharan Africa and only 55% of the demand for contraceptives is satisfied. This is in sharp contrast to 6–8% unmet need and 80% demand satisfied in Europe and Northern America [5, 6].

In 2012 the London Summit on Family Planning launched the Family Planning 2020 (FP2020) initiative to revitalize the global family planning agenda. The initiative aimed to enable an additional 120 million women in using a contraceptive method by the year 2020 [7]. During the summit, Ethiopia made bold commitments to reduce the unmet need to 10% and increase contraceptive prevalence to 55% [8, 9]. Currently in Ethiopia, contraceptive methods are provided for free in public health facilities and through community health workers [10, 11]. Contraceptive utilization among married women has increased sharply from 6% in 2000 to 35% in 2016. However, 22% of currently married women have unmet need [12].

Addressing unmet need and improving access to contraceptives is especially important for women with medical conditions as unintended pregnancy can have serious health consequences [13]. Certain cardiovascular diseases (CVD) are associated with significant obstetric and fetal complications [14]. Despite recent advances in medical and obstetric care, pregnancies complicated by CVD are associated with a high risk of morbidity and mortality [15].

Contraceptive utilization offers a unique opportunity to ensure pregnancy is either avoided or postponed until the woman’s condition is optimized. For those women considering pregnancy, potentially teratogenic drugs can be switched to safer options when possible [16, 17].

Pregnancy among women with CVD was found to have increased the risk of maternal morbidity and mortality as well as adverse pregnancy outcomes [15, 18]. Preventing unintended pregnancy is, therefore, crucial to improve pregnancy outcomes and minimize complications of CVD during pregnancy. However, unmet need for contraceptives continues to undermine the potential benefits of contraceptive use [19]. Studies show that 40–65% of pregnancies among women with CVD are unintended [18, 20] and cardiac illness is one of the most common medical indications for termination of pregnancy [21]. Preexisting cardiac disease is the leading cause of maternal mortality in the developed world. It is also the most common indirect cause of maternal death in low/middle-income countries [22, 23].

Unmet need for contraception has been extensively studied among women in the general population and to some extent in different subpopulations such as women living with HIV [24–26]. However, there are no studies among women with cardiovascular disease. The aim of this study was therefore to determine the rate of unmet need for contraceptives and associated factors among women with cardiovascular disease.

## Methods

### Study design and setting

A facility-based cross-sectional study was conducted at the cardiac clinic of Saint Paul’s Hospital Millennium Medical College (SPHMMC), in Addis Ababa, Ethiopia from February 1 to May 31, 2020. SPHMMC is a tertiary teaching hospital and mainly serves as a public referral center. The cardiac clinic runs two days per week from 8:00 AM to 5: 00 PM and is visited by an average of 40–50 patients per day.

### Populations

Source population- women of reproductive age (15–49 years) with cardiovascular disease who are currently married or in-union attending the cardiac clinic of the hospital.

Study population- sampled women of reproductive age (15–49 years) with cardiovascular disease who are currently married or in-union attending the cardiac clinic of the hospital during the study period.

We excluded women who presented with acute medical conditions and unable to give consent and women who were visiting the cardiac clinic for the first time.

### Sample size and sampling

An initial sample size of 384 was calculated using a single population proportion formula ${n}_{i}={Z}^{2} x P x (1-P)/{d}^{2}$, where *Z* = confidence interval at 95% thus 1.96; and *d* = 5% margin of error. Since no previous study has been done in the setting, the proportion of women with unmet need was assumed to be 50%. This assumption was used as it will yield the largest sample size possible.

Because the total number of women on follow-up was about 1000, a finite population correction was made to the initial sample size.

$$n=\frac{{n}_{i}}{\left(1+\frac{{n}_{i}}{N}\right)}$$

where *n* = adjusted sample size, *n*_*i*_ = the initial sample size, and *N* = total population.

After adding 5% to compensate for non-response, the final sample size was calculated to be 291. Study participants were enrolled consecutively until the sample size was reached.

### Data collection

Data was collected through an exit interview of sampled women using a paper-based, structured, and pretested questionnaire. The questionnaire and the algorithm used to assess unmet needs were developed by referring to various literature [27–29] and the DHS analytical studies definition of unmet need revised in 2012 (Additional file 1 and 2) [30]. The data collection tool was prepared in English and translated into Amharic and translated back to English again to check for consistency. A pre-test was done on 10% (29) participants before data collection. Four trained and experienced data collectors with BSc in nursing were supervised by the principal investigator during the interview.

### Study variables

The dependent variable of the study was unmet need for contraception. The independent variables were socio-demographic characteristics (such as age, level of education, marital status, occupation, and income); previous delivery, counseling on contraceptive use, previous use of a contraceptive method, counseling on risks of unintended pregnancy, partner support on contraceptive use and duration of follow-up at the cardiac clinic.

### Operational definitions

#### Unmet need for contraception

Percentage of women who (1) are not pregnant and not postpartum amenorrhoeic and are considered fecund and want to postpone their next birth for 2 or more years or stop childbearing altogether but are not using a contraceptive method, or (2) have a mistimed or unwanted current pregnancy, or (3) are postpartum amenorrhoeic and their last birth in the last 2 years was mistimed or unwanted.

#### Total demand for contraception

Refers to women with unmet need plus the percentage of women currently using contraception (representing “met need”).

#### Proportion of demand satisfied

The percentage of women using contraception divided by the percentage of women with demand for contraception.

### Data processing and analysis

All the filled questionnaires were verified by the principal investigator. Consecutive code was given to each case and the data was explored again for inconsistencies and missing values. After completeness and coding of questionnaires were checked, data were entered, cleaned, and analyzed using IBM SPSS Statistics for Windows, version 20 (IBM Corp., Armonk, N.Y., USA). Physical data from paper forms were compiled in binders and stored in locked file cabinets whereas electronic data were stored in password-protected computers.

Univariate analyses were carried out to describe the data. The DHS algorithm was applied to assess the unmet need for contraception. A two-stage process was then carried out to identify variables associated with unmet need. Bivariate associations between unmet need and covariates were explored using a Chi-square test. Variables with a *p*-value of less than 0.2 in binary logistic regression were selected as candidates for multivariable logistic regression to control for possible confounding factors. Finally, multivariable logistic regression with odds ratio and 95% confidence interval were computed to assess the presence and strength of association between unmet need and explanatory variables. A *p*-value of less than 0.05 was taken as statically significant.

### Ethical considerations

Ethical clearance for the study was obtained from the Institutional Review Board of SPHMMC before the start of data collection. All participants were informed about the purpose of the study and its procedures. It was made clear to all subjects that participation was voluntary. Privacy and confidentiality of study participants were ensured throughout the study. All participants provided written informed consent. For participants below 18 years of age, consent to participate was obtained from their parents or their legal guardians.

## Results

### Socio-demographic characteristics of study participants

A total of 291 eligible women were approached for the interview. Out of these, 7 were non-responders (5 did not consent to the study, and 2 discontinued the interview) which makes the response rate 97.6%. The majority of the study subjects were between the age of 19–34 years with a mean age of 34 ± 7.4 years. Three fourth of them were married (Table 1).

**Table 1:** Socio-Demographic characteristics of study subjects, Addis Ababa, Ethiopia, 2020

Characteristics	Frequency (n)	Percentage (%)
**Age (years)**		
15–19	7	2.5
20–24	23	8.1
25–29	48	16.9
30–34	78	27.5
35–39	62	21.8
40–44	48	16.9
45–49	18	6.3
**Level of education**		
No formal education	53	18.7
Primary	67	23.6
Secondary	83	29.2
Technical/vocational	23	8.1
Higher	58	20.4
**Marital status**		
Married	214	75.4
In union (living with a man)	70	24.6
**Occupation**		
Student	16	5.6
Unemployed	16	5.6
Housewife	135	47.5
House servant	9	3.2
Daily laborer	20	7.0
Merchant	55	19.4
Government employee	33	11.6
Private employee	16	5.6
Other	16	5.6
**Monthly income (Ethiopian Birr)**		
No monthly income	171	60.2
Less than 1000	7	2.5
1001- 3000	54	19.0
3001–5000	25	8.8
More than 5001	27	9.5

### Sexual and reproductive characteristics of study participants

Among 284 interviewed women, 198 (69.8%) have used a modern contraceptive method at least once in their lifetime. There were 51 pregnant women during the study, of which 25 (49%) had an unintended pregnancy. More than 87% of the participants were sexually active within the past year and close to 70% reported sexual activity in the month preceding the study.

### Cardiac care and follow up

The most commonly observed cardiovascular disease were hypertension 90 (31.7%), chronic rheumatic valve disease 89 (31.3%), and congenital heart disease 47 (16.4%) (Table 2). The duration of chronic care and treatment ranges from 3 months to 20 years and the median duration was 36 months (IQR: 24–73).

**Table 2:** Types of cardiovascular disease among study subjects, Addis Ababa, Ethiopia, 2020

Type of cardiovascular disease	Frequency (N)	Percentage (%)
Hypertension	90	31.7
Chronic rheumatic valve disease	89	31.3
Adult congenital heart disease	47	16.6
Coronary heart disease	26	9.2
Cardiomyopathy	22	7.7
Others	10	3.5
**Total**	**102**	**100**

### Contraceptive counseling

One-third of women discussed the risks of unintended pregnancy and future pregnancy plans with their care providers. In addition, 117 (41.2%) were counseled on contraceptive utilization whereas only 25% were linked with the family planning unit of the hospital.

### Unmet need for contraception and associated factors

**Fig. 1:**
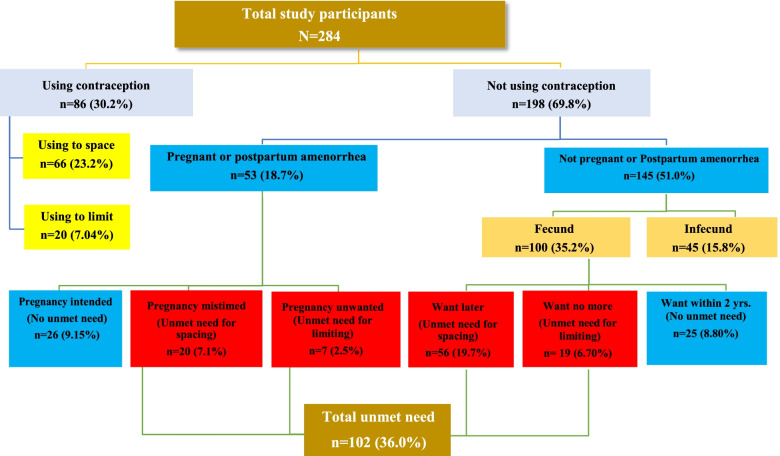
Illustration of calculated unmet need for contraception among study subjects, Addis Ababa, Ethiopia, 2020.

Figure 1 shows the stepwise algorithm used to calculate unmet need for contraception in the sampled population. The overall unmet need for contraception was 36.0% (95% CI: 30.4–41.5), with 26.8% (95% CI: 21.6–32.0) having unmet need for spacing and 9.2% (95% CI: 5.8–12.5) for limiting.

The main reasons for not using contraceptive methods were fear of side effects and drug interactions, not having frequent sex, and not being married (Table 3).

**Table 3:** Reasons for not using contraceptives among women with unmet need, Addis Ababa, Ethiopia, 2020

Reason for not using	Frequency (N)	Percentage (%)
**Perceived low risk of pregnancy**		
No having frequent sex	24	23.5
Not married	18	17.7
Breastfeeding	13	12.8
Up to God/fatalistic	8	7.8
**Method related reasons**		
Fear of drug side effect and interaction	30	29.4
Lack of awareness	3	2.9
**Opposition to use**		
Partner opposing	4	3.9
**Other**	2	2
**Total**	**102**	**100**

**Fig. 2:**
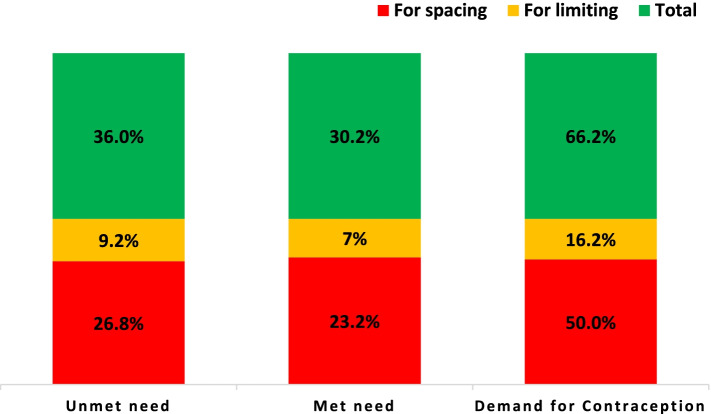
The percentage of unmet need, met need, and demand satisfied for contraception among study subjects, Addis Ababa, Ethiopia, 2020.

The contraceptive utilization (representing the met need) was 30.2% (95% CI: 25.0–36.5). Thus, the demand for contraception was 66.2% (95% CI: 60.7–71.7) and the demand satisfied was 45.6% (95% CI: 39.8–51.4) (Fig. 2).

Bivariate and multivariable logistic regression analyses were done to explore factors associated with unmet need. In bivariate analyses marital status, counseling on contraceptive use, previous use of a contraceptive method, counseling on risks of unintended pregnancy, and partner support on contraceptive use were associated with unmet need. In subsequent multivariable logistic regression marital status, counseling on contraceptive use, previous use of a contraceptive method, and partner support on contraceptive use remained independently associated with unmet need (Table 4).

**Table 4:** Association of independent variables with unmet need for contraception among study subjects, Addis Ababa, Ethiopia, 2020

Characteristics	Unmet need		Crudes OR (95% CI)	Adjusted OR (95%CI)	*P*-value
	**No**	**Yes**			
**Marital status**					0.04
Married	171	43	1.00	1.00	
Living with man	11	59	21.3 (10.3–44.1)	9.4 (2.9–30.6)^a^	
**Counseled on use of contraceptives**					< 0.001
Yes	77	6	1.00	1.00	
No	105	96	11.7 (4.8–28.2)	6.7 (1.8–24.7)^a^	
**Previously used a contraceptive method**					< 0.001
Yes	151	48	1.00	1.00	
No	31	54	5.5 (3.17–9.48)	3.2 (1.12–8.92)^a^	
**Counseled on the risk of unintended pregnancy**					0.06
Yes	71	21	1.00	1.00	
No	102	76	2.52 (1.42–4.46)	0.5 (0.15–1.81)	
Don’t remember	9	5	1.88 (0.57–6.22)	0.8 (0.08–8.14)	
**Partner support on contraceptive use**					< 0.001
Yes	135	45	1.000	1.00	
No	19	49	7.7 (4.13–14.5)	6.2 (1.91–19.8)^a^	
I don’t know	28	8	0.86 (0.36–2.02)	1.3 (0.23–7.71)	

^a^Statically significant *CI* Confidence interval, *OR* Odds ratio

Unmet need for contraception was found to be more likely among those who have not been counseled on contraceptive utilization (AOR = 6.7, 95% CI: 1.8–24.7) and those who lack partner support on contraception use (AOR = 6.2, 95% CI: 1.91–19.8). Unmet need was also found to be more likely among those who have never used a contraceptive method in the past (AOR = 3.2, 95% CI: 1.12–8.92) (Table 4).

Other variables like age, educational status, occupation, income, ever giving birth, and duration of follow-up were not found to be significantly associated with unmet need for contraception.

## Discussion

While several studies evaluated levels of unmet need and associated factors among women in the general population, there is a lack of evidence for women with medical comorbidities. This study aimed to fill the evidence gap about unmet need among women with cardiovascular disease. The findings will enable the design, implementation, and evaluation of tailored interventions to reduce unmet and ultimately improve pregnancy outcomes, and decrease maternal morbidity and mortality.

The overall unmet need for contraception was 36% (95% CI: 30.4–41.5) in this study. This is higher than the figures reported for currently married reproductive age women in the Ethiopian Demographic and Health Survey (EDHS) 2016, 22% at the national level, and 11% in Addis Ababa [12]. Though the settings are different, these discrepancies are particularly important as contraceptive utilization in this particular patient population has a pivotal role to play in reducing maternal morbidity and mortality.

In our study, the demand for contraceptives was 66.2% (95% CI: 60.7–71.7) compared to 58% in the general population. Furthermore, 50% of the demand for contraceptives was for spacing (Fig. 2). This may indicate that women wish to continue procreation despite their medical comorbidity. However, only 45.6% (95% CI: 39.8–51.4) of the demand is met in our study which is much lower than the demand satisfied at the national level (62%) and in Addis Ababa (84%) [12]. These findings underline how significant contraceptive service is for this group of population and the need to design tailored service provision models to address their needs and achieve their reproductive goals.

The rate of contraceptive utilization in our study was low compared to studies from developed countries. Studies done in the USA and Germany showed that 75% of adult women with congenital heart disease were using a method of contraception [20, 31]. In contrast a study done at Gondar University hospital in northern Ethiopia among diabetic and hypertensive patients, the contraceptive use rate was 53.3% [32]. These differences can be explained by differences in socio-demographic characteristics of study participants and institutional variations in contraceptive counseling and service provision.

In this study, the association between variables and unmet need was assessed. Women who have not been counseled on contraceptive utilization were six times more likely to have unmet need. This trend is consistent with findings from other studies which reveal higher rates of unmet need among those who did not receive contraceptive counseling [28, 29, 33]. Importantly the most frequent reasons for contraception non-use among women with unmet need were related to perceived low risk of pregnancy and fear of side effects and drug interaction. These findings indicate the need to incorporate appropriate counseling sessions that inform women of the risk of unintended pregnancy and its impact on their health [2–4].

Most patients with CVD have multiple visits before conception, offering a golden opportunity for contraceptive counseling [15]. The World Health Organization provides a clinical guide to aid contraceptive provision for women with medical conditions [34]. This tool has been modified to further stratify the risk of pregnancy in CVD and provide tailored counseling [16]. A multidisciplinary team composed of cardiologists, family planning, and maternal–fetal medicine specialists can deliver individualized counseling on contraception and pregnancy [16, 35]. These sessions increase women’s contraceptive knowledge, address their concerns and fears, and clear misconceptions about contraception. These coordinated efforts will also ensure women are provided with contraceptive methods that are effective and safe for their particular condition and can serve as a gateway for other reproductive health services other than family planning [35, 36].

Women who have never used a contraceptive method in the past were more likely to have unmet need. This is consistent with findings from other studies across different settings [27, 37, 38]. A possible explanation for this finding is that previous users are more likely to be well informed and experienced on potential adverse effects of contraceptive methods. This suggests that, once a woman has tried contraception methods, she may be more likely to continue using it provided that other barriers are addressed [39].

Having partner support on contraception use was another important factor associated with unmet need for contraception. Thus, strategies that engage male partners in contraceptive counseling sessions and foster open discussion among couples while respecting woman’s autonomy can improve contraceptive utilization [40, 41].

The strength of this study includes the utilization of a standardized and validated DHS definition of unmet need. Though the algorithm was initially designed for women in the general population, it has been widely implemented across different subpopulations. Furthermore, this tool allows valid comparison across settings and over time [30]. Our study provides evidence from a large tertiary referral facility in a low-income country. As such, the results can be used to compare findings and plan interventions in other institutions with similar settings. Thus, the findings from this study can serve as a benchmark to track the impacts of future interventions and strategies at this and other similar facilities.

Due to limited resources, this study did not examine all variables that may affect unmet need including provider factors related to contraceptive counseling or referral practice which can influence unmet need. Thus, further research that explores the knowledge, attitude, and practice of providers is warranted.

## Conclusion

The study revealed a high rate of unmet need for contraception among women with CVD. Marital status, previous use of a contraceptive method, counseling on contraceptive use, and partner support on contraceptive use were independent predictors of unmet need. The cardiac follow-up clinic should implement client-centered counseling by a multidisciplinary team to address the needs of women and prevent consequences of unintended pregnancy. Furthermore, there is a need to initiate interventions that encourage communication between couples and increase male partner involvement through a renewed focus on couples counseling.

## Supplementary Information

**Additional file 1.** Assessment of unmet need.

**Additional file 2.** Algorithm_ Revised definition.

## Data Availability

The datasets used and/or analyzed during the current study are available from the corresponding author on reasonable request.

## Abbreviations

AOR: Adjusted Odds Ratio
CI: Confidence Interval
CVD: Cardiovascular Disease
EDHS: Ethiopia Demographic and Health Survey
MEC: Medical Eligibility Criteria
SPHMMC: Saint Paul’s Hospital Millennium Medical College
WHO: World Health Organization
