# Supplementary material for: Unmet need for contraception and associated factors among women with cardiovascular disease having follow-up at Saint Paul’s Hospital Millennium Medical College, Addis Ababa, Ethiopia: a cross-sectional study
Source: Contracept Reprod Med. 2022 May 11;7:6. doi: 10.1186/s40834-022-00173-0 (PMC9092812; doi:10.1186/s40834-022-00173-0)
Supplement: Supplementary file 1 — Additional file 1. Assessment of unmet need. [file 40834_2022_173_MOESM1_ESM.docx]

**Assessment of unmet need**

| **No.** | QUESTIONS AND FILTERS | CODING CATEGORIES | **SKIP** |
| --- | --- | --- | --- |
| **226** | Are you pregnant now? | YES . . . . . . . . . . . . . . . . . . . . . . . . . . . . 1  NO. . . . . . . . . . . . . . . . . . . . . . . . . . . . . .2  UNSURE. . . . . . . . . . . . . . . . . . . . . . . . 8 | **238** |
| **228** | When you got pregnant, did you want to get pregnant at that time? | YES . . . . . . . . . . . . . . . . . . . . . . . . . . . . 1  NO. . . . . . . . . . . . . . . . . . . . . . . . . . . . . .2 | **238** |
| **229** | Did you want to have a baby later on or did you not want any (more) children? | LATER. . . . . . . . . . . . . . . . . . . . . . . . . . 1  NO MORE . . . . . . . . . . . . . . . . . . . . . . .2 |  |
| **238** | When did your last menstrual period start?  ____________________  (DATE, IF GIVEN) | \|  \|  \| \| --- \| --- \| \|  \|  \| \|  \|  \| \|  \|  \|   DAYS AGO . . . . . . . . . . . . 1  WEEKS AGO . . . . . . . . . . .2  MONTHS AGO. . . . . . . . . .3  YEARS AGO . . . . . . . . . . . 4  IN MENOPAUSE/  HAS HAD HYSTERECTOMY. . 994  BEFORE LAST BIRTH. . . . . . . . . . 995  NEVER MENSTRUATED. . . . . . . . 996 |  |
| **302** | CHECK 226:  PREGNANT  NOT PREGNANT OR UNSURE | | **313** |
| **303** | Are you currently doing something or using any method to delay or avoid getting pregnant? | YES. . . . . . . . . . . . . . . . . . . . . . . . . . . . .1  NO . . . . . . . . . . . . . . . . . . . . . . . . . . . . . 2 |  |
| **313** | Have you ever used anything or tried in any way to delay or avoid getting pregnant? | YES. . . . . . . . . . . . . . . . . . . . . . . . . . . . .1  NO . . . . . . . . . . . . . . . . . . . . . . . . . . . . . 2 |  |
| **501** | Have you ever given birth? | YES. . . . . . . . . . . . . . . . . . . . . . . . . . . . .1  NO . . . . . . . . . . . . . . . . . . . . . . . . . . . . . 2 | **601** |
| **No.** | QUESTIONS AND FILTERS | CODING CATEGORIES | **SKIP** |
| **212** | What name was given to your (last) baby?  RECORD NAME | NAME ________________________ |  |
| **215** | In what month and year was (NAME) born?  PROBE:  When is his/her birthday? | \|  \|  \| \| --- \| --- \|   MONTH . . . . . . . . . . . . . . . . .   \|  \|  \|  \|  \| \| --- \| --- \| --- \| --- \|   YEAR . . . . . . . . . . . . |  |
| **401** | CHECK 215:  BIRTH BEFORE 2005  BIRTH IN 2005 OR LATER | | **601** |
| **405** | When you got pregnant with (NAME), did you want to get pregnant at that time? | YES. . . . . . . . . . . . . . . . . . . . . . . . . . . . .1  NO . . . . . . . . . . . . . . . . . . . . . . . . . . . . . 2 | **447** |
| **406** | Did you want to have a baby later on, or did you not want any (more) children? | LATER . . . . . . . . . . . . . . . . . . . . . . . . . .1  NO MORE. . . . . . . . . . . . . . . . . . . . . . . 2 |  |
| **447** | Has your menstrual period returned since the birth of (NAME)? | YES . . . . . . . . . . . . . . . . . . . . . . . . . . . .1  NO . . . . .. . . . . . . . . . . . . . . . . . . . . . . . .2 |  |
| **601** | Are you currently married or living together with a man as if married? | YES, CURRENTLY MARRIED. . . . . .1  YES, LIVING WITH A MAN. . . . . . . .2 |  |
| **610** | Now I would like to ask about your (first) (husband/partner). In what month and year did you start living with him? | \|  \|  \| \| --- \| --- \|   MONTH . . . . . . . . . . . . . . . . .  DON'T KNOW MONTH. . . . . . . . . . .98   \|  \|  \|  \|  \| \| --- \| --- \| --- \| --- \|   YEAR . . . . . . . . . . . .  DON'T KNOW YEAR. . . . . . . . . . .9998 |  |
| **No.** | QUESTIONS AND FILTERS | CODING CATEGORIES | **SKIP** |
| **615** | When was the last time you had sexual intercourse?   - IF LESS THAN 12 MONTHS, ANSWER MUST BE RECORDED IN DAYS, WEEKS OR MONTHS. - IF 12 MONTHS (ONE YEAR) OR MORE, ANSWER MUST BE RECORDED IN YEARS. | \|  \|  \| \| --- \| --- \| \|  \|  \| \|  \|  \| \|  \|  \|   DAYS AGO . . . . . . . . . . . . 1  WEEKS AGO . . . . . . . . . . .2  MONTHS AGO. . . . . . . . . .3  YEARS AGO . . . . . . . . . . . 4 |  |
| **702** | CHECK 226:  NOT PREGNANT OR UNSURE  PREGNANT | | **704** |
| **703** | Now I have some questions about the future. After the child you are expecting now, would you like to have another child, or would you prefer not to have any more children? | HAVE ANOTHER CHILD . . . . . . . . . .1  NO MORE . . . . . . . . . . . . . . . . . . . . . . 2  UNDECIDED/DON'T KNOW. . . . . . . .8 | **705**  **END** |
| **704** | Now I have some questions about the future. Would you like to have (a/another) child, or would you prefer not to have any (more) children? | HAVE (A/ANOTHER) CHILD . . . . . . 1  NO MORE/NONE . . . . . . . . . . . . . . . . .2  SAYS SHE CAN'T GET PREGNANT. 3  UNDECIDED/DON'T KNOW . . . . . . . 8 | **707**  **END** |
| **705**  How long would you like to wait from now before the birth of (a/another) child? | CHECK 226:  PREGNANT  NOT PREGNANT  OR UNSURE  After the birth of the child you are expecting now, how long would you like to wait before the birth of another child? | \|  \|  \| \| --- \| --- \| \|  \|  \|   MONTHS. . . . . . . . . . . . . . .1  YEARS. . . . . . . . . . . . . . . . .2  SOON/NOW…………. . . . . . . . . . ...993  SAYS SHE CAN'T GET PREGNANT………………………...994  AFTER MARRIAGE. . . . . . . . . . . . .995  OTHER ______________________ 996  (SPECIFY)  DON'T KNOW . . . . . . . . . . . . . . . . . 998 | **END END**  **END** |
| **No.** | QUESTIONS AND FILTERS | CODING CATEGORIES | **SKIP** |
| **706** | CHECK 226:  NOT PREGNANT OR UNSURE  PREGNANGT | | **END** |
| **707** | CHECK 303: USING A CONTRACEPTIVE METHOD?  CURRENTLY USING  NOT CURRENTLY USING | | **END** |
| **708**  NOT ASKED | CHECK 705:  00-23 MONTHS  OR 00-01 YEAR  24 OR MORE MONTHS OR 02 OR MORE YEARS | | **END** |
| **709**  WANTS TO HAVE A/ANOTHER CHILD | CHECK 704:  WANTS NO MORE/NONE  You have said that you do not want any (more) children.  Can you tell me why you are not using a method to prevent pregnancy?  Any other reason?  You have said that you do not want (a/another) child soon.  Can you tell me why you are not using a method to prevent pregnancy?  Any other reason? | NOT MARRIED. . . . . . . . . . . . . . . . . . . . . .1    FERTILITY-RELATED REASONS  NOT HAVING SEX . . . . . . . . . . . . . . . . . . . 2  INFREQUENT SEX . . . . . . . . . . . . . . . . . . . 3  MENOPAUSAL/HYSTERECTOMY. . . . . . .4  CAN'T GET PREGNANT. . . . . . . . . . . . . . . 5  NOT MENSTRUATED SINCE LAST BIRTH . . . . . . . . . . . . . . . . . . . . . . . . . . . . . . 6  BREASTFEEDING. . . . . . . . . . . . . . . . . . . . 7  UP TO GOD/FATALISTIC. . . . . . . . . . . . . . .8    OPPOSITION TO USE  RESPONDENT OPPOSED. . . . . . . . . . . . . . . .9  PARTNER OPPOSED. . . . . . . . . . . . . . . . . 10  OTHRS OPPOSED. . . . . . . . . . . . . . . . . . . 11  RELIGIOUS PROHIBITION. . . . . . . . . . . . 12    LACK OF KNOWLEDGE  KNOWS NO METHOD . . . . . . . . . . . . . . . . .13  KNOWS NO SOURCE. . . . . . . . . . . . . . . . . . .14    METHOD-RELATED REASONS  SIDE EFFECTS/HEALTH CONCERNS. . . . . . . . . . . . . . . . . . . . . . . . . 15  LACK OF ACCESS/TOO FAR. . . . . . . . . . 16  COSTS TOO MUCH. . . . . . . . . . . . . . . . . . 17  PREFERRED METHOD NOT AVAILABLE . . . . . . . . . . . . . . . . . . . . . . . . 18  NO METHOD AVAILABLE. . . . . . . . . . . . 19  INCONVENIENT TO USE. . . . . . . . . . . . . 20  OTHER (SPECIFY)_______________21 DON'T KNOW. . . . . . . . . . . . . . . . . . . . . . .98 |  |
