# Supplementary material for: Unmet need for contraception and associated factors among women with cardiovascular disease having follow-up at Saint Paul’s Hospital Millennium Medical College, Addis Ababa, Ethiopia: a cross-sectional study
Source: Contracept Reprod Med. 2022 May 11;7:6. doi: 10.1186/s40834-022-00173-0 (PMC9092812; doi:10.1186/s40834-022-00173-0)
Supplement: Supplementary file 2 — Additional file 2. Algorithm_ Revised definition. [file 40834_2022_173_MOESM2_ESM.docx]

Revised definition of unmet need, currently married women. (Bradley, Croft, Fishel, and Westoff, 2012. Revising Unmet Need for Family Planning)
